# Relationship between Chlorophyll a Concentration, Light Attenuation and Diving Depth of the Southern Elephant Seal *Mirounga leonina*

**DOI:** 10.1371/journal.pone.0047444

**Published:** 2012-10-17

**Authors:** Thomas Jaud, Anne-Cécile Dragon, Jade Vacquie Garcia, Christophe Guinet

**Affiliations:** Marine Predator Department, Centre Biologique de Chizé, Villiers en Bois, France; Texas A&M University-Corpus Christi, United States of America

## Abstract

Recently, a number of Antarctic marine environmental studies have used oceanographic parameters collected from instrumented top predators for ecological and physical information. Phytoplankton concentration is generally quantified through active measurement of chlorophyll fluorescence. In this study, light absorption coefficient (K_0.75_) was used as an indicator of phytoplankton concentration. This measurement, easy to obtain and requiring low electric power, allows for assessing of the fine scale horizontal structuring of phytoplankton. As part of this study, Southern elephant seals (SES) were simultaneously equipped with a fluorometer and a light logger. Along the SES tracks, variations in K_0.75_ were strongly correlated with chlorophyll, a concentration measured by the fluorometer within the euphotic layer. With regards to SES foraging behaviour, bottom depth of the seal’s dive was highly dependent on light intensity at 150 m, indicating that the vertical distribution of SES’s prey such as myctophids is tightly related to light level. Therefore, change in phytoplankton concentration may not only have a direct effect on SES’s prey abundance but may also determine their vertical accessibility with likely consequences on SES foraging efficiency.

## Introduction

Within the context of a rapidly changing climate, it is essential to understand how both environment and species will respond to such changes over time. In marine ecosystems, such investigations are challenging due to their high spatio-temporal dynamics and the fact that these changes occur in three dimensions. As a result, collection of data is necessary throughout the water column, and requires adapted and often costly observation methods (cruise, Argo float, drifter, gliders…). This is particularly true for the Southern Ocean (SO) due to its remoteness and harsh weather conditions.

New technologies such as ARGO floats have considerably increased the amount of data available [1], [2]. However, in the Southern Ocean, these floats are advected eastward by the strong Antarctic Circumpolar Current and the presence of sea-ice prevents homogeneous sampling [3]. Satellite coverage provides surface observations of a broad range ofoceanographic parameters. However, the collection of remotely-sensed sea surface temperature (SST) and surface chlorophyll a (chl-a)concentration are often limited within the SO due to extensive cloud cover. Therefore, data on SST or surface chl-a needs to be merged over several days or weeks to provide a synoptic view of conditions. This loss of temporal resolution induces a concomitant loss in the spatial resolution of the oceanographic features structuring the SO.

Over the last two decades, several studies have quantified the foraging distributions of top marine predators using Satellite Data Relayed Loggers (SRDLs) [4]. Tagged animals have included sharks [5], [6], sea birds [7] and marine mammals [8]–[10]. Increasingly, certain top predator species have become major sources of oceanographic data [2], [11]–[13]. The SEAOS (Southern Elephant Seals as Oceanographic Sampler) and MEOP (Marine Mammal Explorer of the Ocean from Pole to Pole) [14] programs implemented a new generation of oceanographic SRDLs developed by the Sea Mammal Research Unit (SMRU) in collaboration with several research institutions. These are probably the best examples of how marine predators can complement observation systems, with southern elephant seals (SES) currently providing 98% of the temperature and salinity profiles available within the Antarctic pack ice zone.

Due to their at-sea ecology, SES are ideal platforms to collect oceanographic data during their extensive foraging migrations [15]. While at sea they spend about 90% of their time under water,dive deep (up to 2000 m) and continuously perform an average of 60 dives per day, with an inter dive surface interval of approximately 3 minutes [2], [16]. Sexual and ontogenetic differences have been observed in foraging strategies [17]–[19]. Sub-adult and adult males forage close to the continental slope (Kerguelen or Antarctic) unlike females who favour oceanic domains [20] such as the polar frontal zone, the Antarctic divergence and the marginal ice zone. Recent isotopic studies have suggested that the female SES’s diet is dominated by lantern fish (i.e. myctophids, [21]).

However, irrespective of these differences, environmental conditions such as temperature [8], [22], sea ice [20], front position [23], water colour [24], [25]and bathymetry have been found to correlate with the diving behaviour of SES.

In order to assess the environmental variable chlorophyll a concentration, fluorometers were recently addedby our research team to the temperature and conductivity sensors on board SMRU’s SRDLs. These CTD-Fluo SRDLs have been deployed on SES since late 2007 and work has been conducted as part of the program “**I**nvestigation of the vulnerability of the biological **P**roductivity of the **S**outhern **O**cean **S**ubsystems to climate change: the **S**outhern **E**lephant seal **A**ssessment from mid to high **L**atitudes” (IPSOS-SEAL). There were three objectives to this project; first to sample fluorescence profiles with Kerguelen’s SES; then assess seasonal and inter-annual changes in primary production of the main oceanographic domains; and finally to study foraging habitats used by Kerguelen’s SES - namely the Kerguelen-Heard plateau, the Antarctic shelf, the Polar frontal zone and the marginal Ice zone [19]. However, due to power supply limitations and reduced Argos bandwidth, only 1 to 3 CTD Fluorescence profiles can be transmitted daily. Furthermore, each profile has to be summarized to the 18 most relevant points to reduce the amount of data before transmission [26]. To overcome these limitations and obtain as high as possible temporal resolution data sets, pressure, temperature and light loggers (MK9 Wildlife computer) were added to the CTD-Fluo SRDLs. This allowed for the continuous recording of these parameters throughout the entire foraging trip of the SES. In a previous study, Teo et al [27] used high temporal resolution light measurements collected by Pacific bluefin Tuna (*Thunnus orientalis*) that had been equipped with light sensors to reconstruct chl-a vertical distribution.

Light is a critical factor controlling the vertical distribution of many marine organisms, ranging from zooplankton [28] to fish [29] and marine mammals [30], which have been shown to distribute themselves according to precise light isolines. Myctophids exhibit nycthemeral migrations [31], coming closer to the surface at night and remaining at greater depths during the day. At the same time, SES exhibit strong variations in dive depth between day and night.

Within the euphotic layer, light is known to be attenuated in relation to the concentration of inorganic and organic particles suspended within the water column. In pelagic waters, phytoplankton constitute the main source of particles in suspension within the euphotic layer and have been shown to be the main cause of light attenuation [32]. In a previous study, Dragon et al. [25] showed that the diving depth of SES was reduced in areas of high surface chl-a concentration, as assessed byocean colour satellites. This result was interpreted as a behavioural response of SES to the shallower distribution of their main myctophid prey species due to a greater attenuation of light under high phytoplankton concentrations.

According to these previous findings, and thanks to the development of a new generation of CTD-Fluo SRDLs, this study had two main objectives. First, using concomitant measurements of light and fluorescence, we wanted to determine whether light attenuation within the euphotic layer of the pelagic SO was directly related to phytoplankton concentration, assessed independently by the *in situ* fluorescence profiles. The second objective of this work was to assess if the diving depth of elephant seals was directly related to light level within the euphotic layers during daylight hours. Our hypothesis here was that SES were diving to shallower depths when light attenuation (i.e. phytoplankton concentration) was greater.

## Materials and Methods

### 1 Deployment

**Figure 1:**
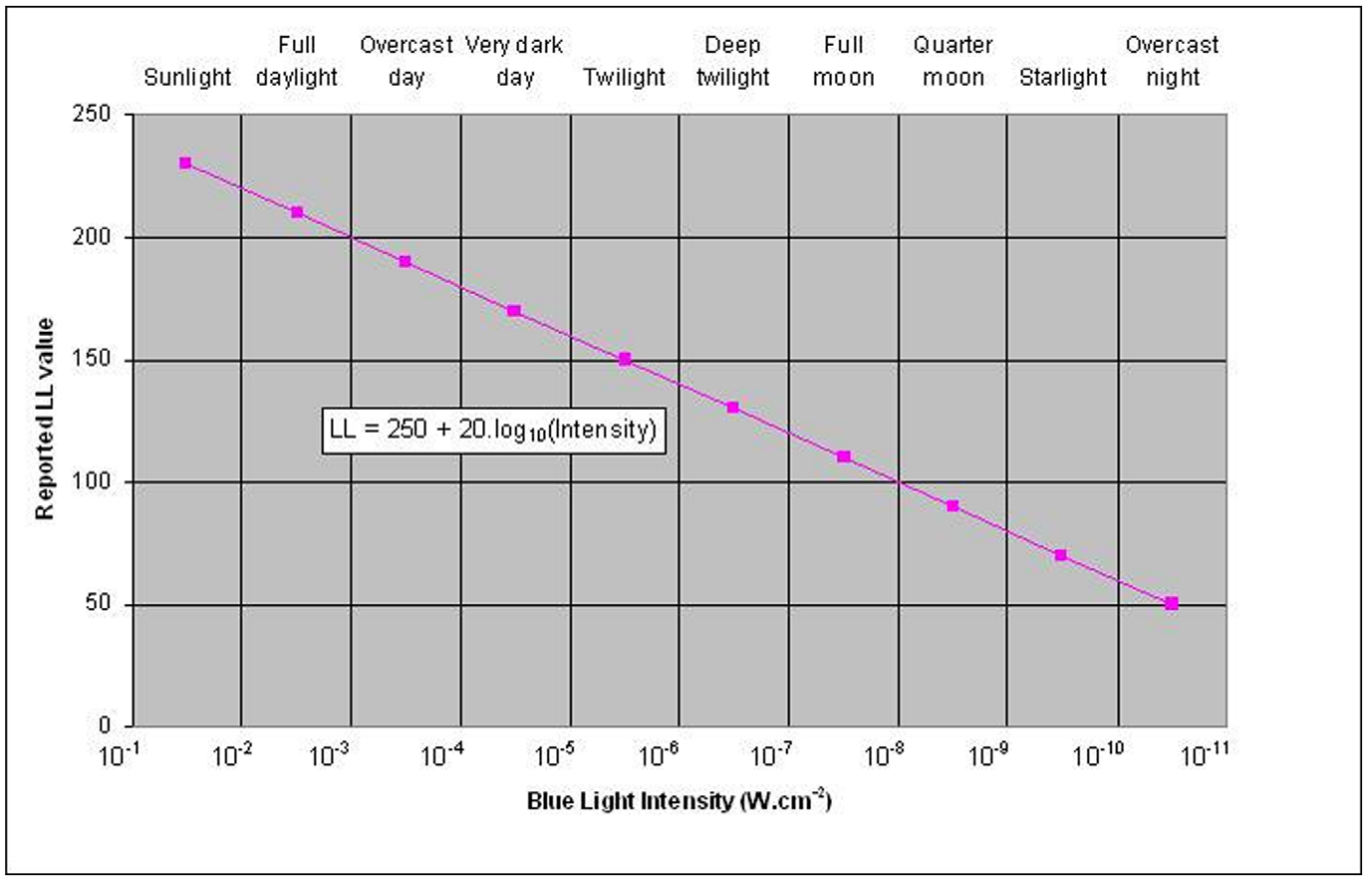
Logarithmic conversion of the Blue light intensity measured by the mk9 logger (Wildlife Computers, Scotland) to Reported LL values. Both axes are divided in different class of light intensity representing different sun conditions.

In October 2009, four post-breeding female SES weighing on average 275.4±19.7 kg for a mean length of 2.3±0.1 m were anesthetised with an intravenous injection of tiletamine and zolazepam 1∶1. Each female was equipped with a CTD-Fluo SRDL (Sea Mammal Research Unit, St Andrews University, Scotland) (SMRU) combined with an MK9 (Wildlife Computer, USA) time depth recorder (TDR) glued on the back of the CTD-Fluo SRDL. The package was then glued to the fur of the SES’s head using a two component industrial epoxy (Araldite AW 2101). The CTD-Fluo SRDL included a Keller type pressure sensor (series PA7 0 to 2000 dbar ±1 dbar), a fast response Platinum Resistance Thermometer (PRT) (−5°C to 35°C ±0.005°C, 0.7 seconds response time), an induction conductivity sensor developed by Valport (UK, range: 0 to 80 mS cm−1, accuracy: better than 0.02 mS cm−1), and a Cyclops 7 fluorometer from Turner Design with a dynamical range set between 0 to 5 µg of chla (chl-a.L^−1^) [14]. The MK9 TDR loggers were set to sample depth (0 to 1500±1 meter), water temperature (−40°C to +60°C ±0.1°C) and light (5.10^−2^ W.cm^−2^ to 5×10^−12^ W.cm^−2^ in blue Wavelength) every two seconds. Light values are converted onboard via a log treatment (Fig. 1) to compress the light measurements to a 3 digit value.

### 2 Fluorescence Data Pre-treatment

Before deployment on SES, each CTD-Fluo SRDL was calibrated at sea during the BOUSSOLE campaign (D. Antoine, Laboratoire Océanographique de Villefranche sur Mer) by comparison with *in situ* measurement from Niskin bottles. A coefficient was calculated for each tag to convert the fluorescence values to an actual chl-a concentration.

When CTD-Fluo SRDLs were deployed on SES, chl-a concentration was assessed continuously at a two second sampling rate during the last 180 m of the ascent phase of the dive, while temperature and conductivity were sampled throughout the diving range. 180 m was selected as the threshold because it encompasses the euphotic layer, which is generally close to 150 m. The mean chl-a concentration was calculated for 10 meter bins, and these values were transmitted via Argos. Temperature and salinity were treated similarly for the first 180 m, plus six measurements were made at depths exceeding 180 to ensure that the best reconstruction of the high resolution temperature and salinity profile to also be transmitted (see [26] for details).

Daylight fluorescence profiles are known to be affected by quenching (i.e. photo-inhibition due to an excess of light) resulting in an artificial deep maximum chl-a concentration. In well mixed waters representing about 84% of all available profiles, the quenching effect was corrected according to the method proposed by ([33]). Daylight profiles obtained in well stratified water (less than 20%) were excluded from the analyses as quenching could not be corrected accurately under these conditions [33]).

Finally for each profile we calculated the summed chl-a value (in µg.L^−1^.) by adding up the 18 chl-a concentration values measured for the corresponding profile.

### 3 Attenuation Coefficient Calculation

The absolute light values are highly influenced by meteorological conditions such as cloud cover or sun incidence angle. As the chl-a concentration was assessed during the ascent phase of the dive, the light attenuation was also calculated during the end of the ascending phase of the dive. Therefore, mean surface light (**IR (0))** was measured for each dive when the seal was near the surface (at depths ranging between 0 to 10 m) during the inter-dive surface interval following the end of the dive. Taking the light reading just below the surface reduces the effect of sun incidence angle in light penetration within the water column.

The relative light values (I(z)) were calculated as.
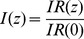
(1)


The Beer Lambert law describes the light attenuation in liquid layers and is used for oceanic water masses [34]. The difference between several wavelengths (**λ**) was not studied here. The attenuation coefficients (k) were calculated accordingly for the end of each ascent phase of a dive during daylight hours with light intensity (L) and depth (z) according to:
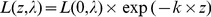
(2)


The quartiles 0, 0.5, 0.75 and 1 of k distributionsweretested to reconstruct the light profiles, using **eq. 2**, and each reconstruction was compared to the actual measurement. The r-squared value between the two distributions and the normalized residuals were calculated and presented in *Table 1*. The quartile 0.75 (**K_0.75_**) provided the best result and was therefore chosen to characterise light attenuation for a dive. Night data was filtered out according to the time and location of the seal using the “sunrise” and “sunset” function of the R package “maptools”.

**Table 1:** Column 1 list the quantile of k0.75 tested to reconstruct light profile, column 2 and 3 the correlation coefficient and residuals proportions of the difference between light intensity observed and calculated, outside the confidence intervals 95%.

Quantile of k	r^2^	Proportion of values outside the confidence interval
0	0.83	4.6
0.5	0.93	4.7
0.75	0.94	4.7
1	0.90	4.8

### 4 Light at 150 m (IR150)

The depth at 150 m is often used as the mean depth of euphotic zone and light level at this threshold was also extracted from the dataset. The light at 150 m was used here as indicator of the light intensity available just under the euphotic (phytoplankton) layer. This value integrates all the factors affecting light attenuation such as the organic and inorganic particle concentration within the euphotic layer, as well as cloud cover and sun angle. This value can easily be extracted from the dataset and marine organisms are likely to react directly to the actual light level rather than to light attenuation in the water column.

### 5 SES’sDiving Bottom Depth (DBD)

The animal vertical movement allows us to measure the depth at which the SES are suspected to forage. The DBD corresponds to the mean depth where the animal is at the deepest phase of the dive (more than 80% of the maximum depth of this dive).

### 6 Data Selection

In order to limit the influence of non-phytoplanktonic particles on light attenuation, turbid continental shelf waters were excluded from the analysis. Only light and fluorescence profiles obtained off the Kerguelen and Crozet shelves (i.e. for isobaths greater than 1000 m) were included in the analyses.

Fluorescence profiles collected during daylight hours were matched with the corresponding ascending light profile according to the time reference.

### 7 Statistical Analysis

The software R [35]with the package “lmer” was used to fit a linear mixed-effects model in the formulation described in Laird and Ware (1982, [36]) but allowing for nested random effects.The individual’s identification number [6]was coded as a random effect to account for the spatial and individual dependence structure between observations. Dates (in days) were also coded as a random effect to account for any seasonal effect on primary production. A range of variables were tested as fixed effect to explain variation in light attenuation and light level at 150 m, and they are described below:

- The bathymetry (**bathy**) is known to be linked to terrestrial particle concentration in ocean water and, thereby, to turbidity.
- The surface light level (**IR_0_**) directly influences light level in deeper water.
- The temperature measured at 200 m is used as an indicator of the animal position relative to the position of the polar front [15].
- The number of temperature inversions, or water masses temperature heterogeneity (**WMTH**), is used as indicator of external water mass incursion and the proximity of a frontal zone [37]. Here an inversion is detected when the difference in temperature between two successive measurements reverses sign.
- The mixed-layer depth (**MLD**) is one of the main factors affecting the vertical distribution of phytoplankton [38], [39].


- Summed Chl-a and DBD were tested against all variables listed above, adding up to **K_0.75_** and **IR150, respectively**.

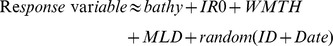



Model selection was performed in three steps. The first was to build a model including all fixed effects available, and then a step-wise procedure was implemented to select the most significant variables. Finally, the best model was selected as the model which had the lowest Bayesian Information Criterion (BIC). The decay of light in the water is described by an exponential law linking the light intensity, the depth and the attenuation coefficient [40]. This coefficient depends on water's inherent optical properties such as the chl-a concentration. A logarithmic relationship between the light attenuation coefficient and the chl-a concentration was implemented in the model. The normality of the residuals was checked graphically and the fitted values of the model were plotted against the observations.

The following hypotheses were tested:

- Summed chl-a values are positively correlated to light attenuation coefficient.
- Diving depths of the SES are positively correlated to light intensity at 150 m and light absorption coefficient.

## Results

The four female SES foraged within the polar frontal zone delineated by the subantarctic and the Southern Antarctic Circumpolar Current fronts. Each CTD-Fluo SRDL provided a daily average of 2.1±0.8 chl-a profiles. Temperature and salinity data were corrected according to the procedure proposed by Roquet et al. ([26])and are stored at the French National Museum of Natural History and in the CORIOLIS data centre (IFREMER-Brest).

**Figure 2:**
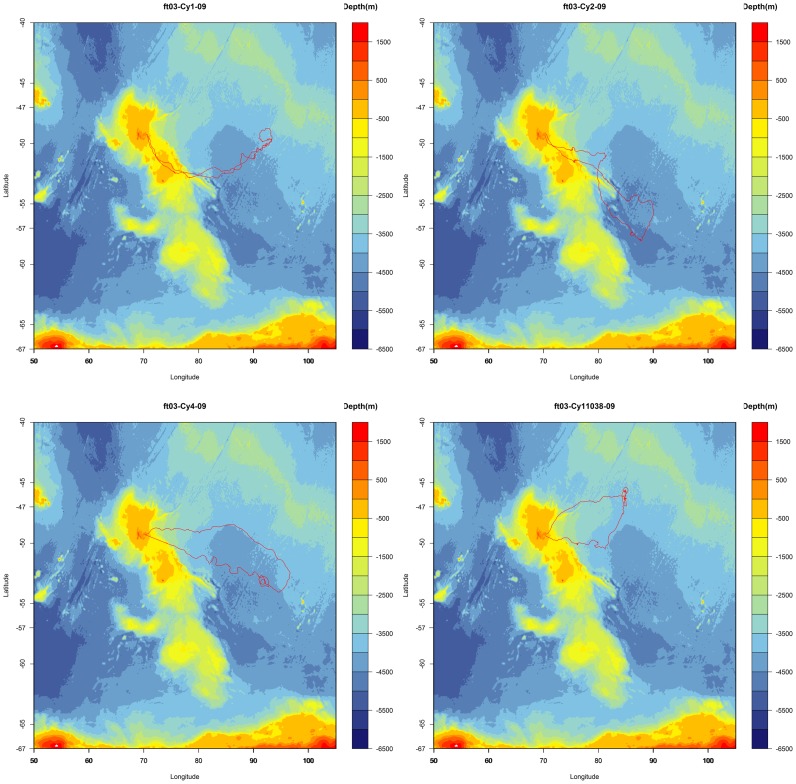
Trajectory of the four seals tagged in October 2009. ft03-Cy1-09, ft03-Cy2-09, ft03-Cy4-09, ft03-Cy5-09.

The four elephant seals performed a total of 21270 dives between the 19th October 2009 and the 1^st^ January 2010 with a mean duration of 18.9±4.9 minutes. The deepest dive reached 1327 m and the longest one lasted 62 minutes. A total of 429 chl-a profiles were transmitted and the trajectories of each seal are shown in Figure 2. After removing the profiles collected over the shelf and during the night, a total of 9742 light and 181 chl-a profiles were available for the analyses. The inboard logarithm transformation of the light values forced us to use an exponential transformation on K_0.75_ values to obtain the real light attenuation coefficient. After correction the mean K_0.75_ value for all seals was 0.0317±0.017 m^−1^.

The correlation between K_0.75_ and the date was tested and found to be significant (r^2^ = 0.88, n = 8312, p-value <0.001), confirming the usefulness of a mixed effect model approach.

### 1 Relationship between Summed chl-a Concentration and Light Attenuation Coefficient

**Figure 3:**
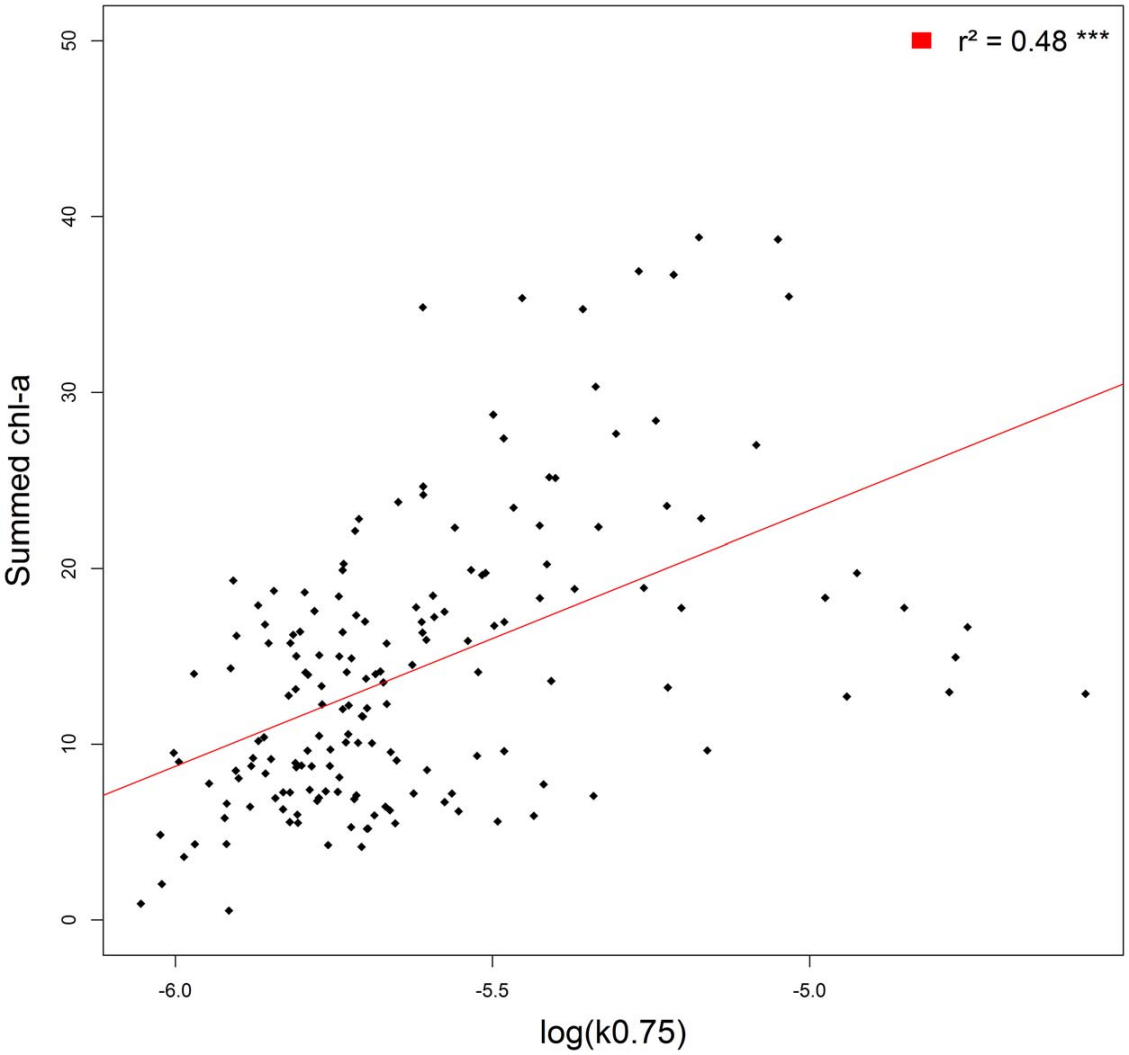
Relationship between the light attenuation coefficient and chlorophyll a concentration. Observations (black) are stack to linear regression line (red). The regression coefficient (r^2^) between the regression line and the observation is noted on the top right corner of the figure.

The model with light attenuation as the only fixed effect was retained after a BIC model selection procedure. As expected, log transformed “summed chl-a concentration” was positively related (estimated coefficient  = 14.67±1.73, p-value <0.001) to the light absorption coefficient. The normality of residuals was graphically assessed. The model showed a positive correlation (r^2^ = 0.41) with observed values (Fig. 3).

### 2 Relationship between Diving Depth and Summed chl-a Concentration

**Figure 4:**
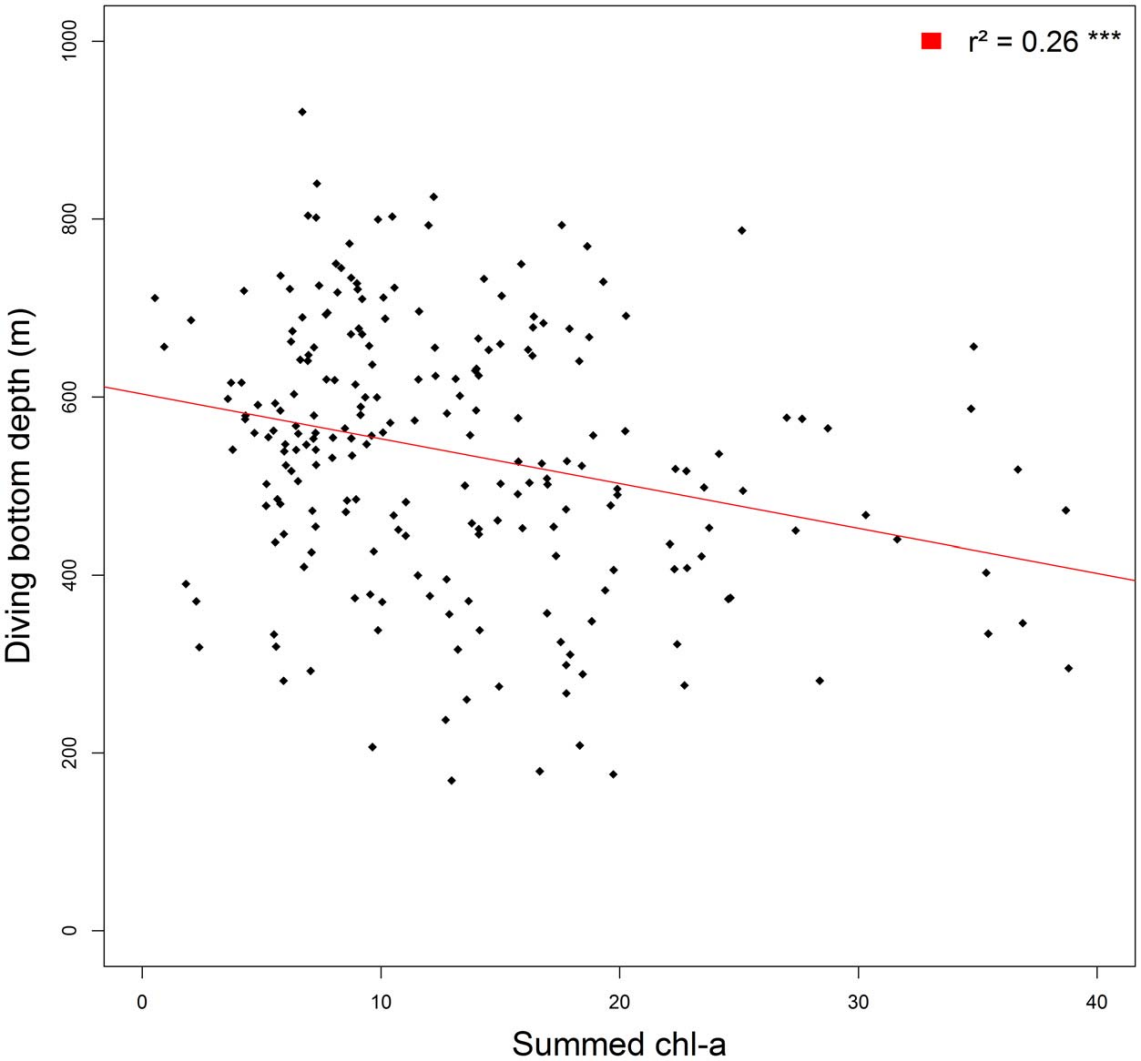
Relationship between summed chlorophyll a concentration and the mean depth of the bottom dive of elephant seals. Observations (black) are stack to linear regression line (red). The regression coefficient (r^2^) between the regression line and the observation is noted on the top right corner of the figure.

After running the BIC selection procedure, DBD was found to be positively related to temperature at 200 m and surface light intensity, but negatively related to summed chl-a concentration (see *Table 2* and Fig. 4).

**Table 2:** Parameters estimation for the second model (DBD ∼ summed chl-a concentration + IR_0_+ temp200) with standard error and p-values.

	Estimated coefficient	Standard error	p-value
Summed chl-aconcentration	− 5.88	1.33	<0.001
Surface light (IR_0_)	2.25	1.12	0.02
Temperature at 200 m	11.01	6.1	0.03

(DBD: Diving Bottom Depth.).

**Figure 5:**
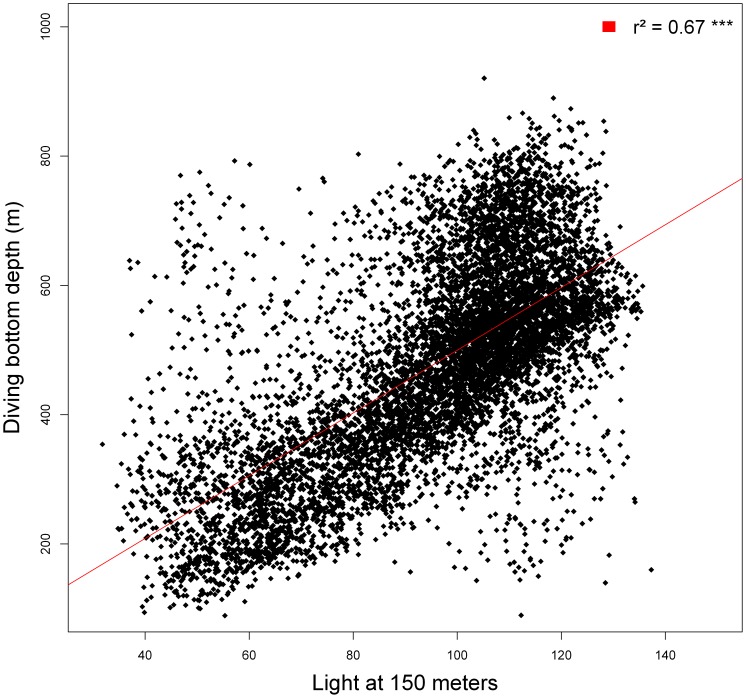
Relationship between the light intensity at 150 m and the mean bottom depth of elephants seals dive. Observations (black) are stack to linear regression line (red). The regression coefficient (r^2^) between the regression line and the observation is noted on the top right corner of the figure.

During daylight hours only, the DBD was found to be strongly and positively related to the light intensity at 150 m (Fig. 5). Furthermore, DBD was also positively related to temperature at 200 m and the water column temperature heterogeneity (see *Table 3*). Temperature heterogeneity and light intensity at 150 m were also retained for night periods (see *Table 4*). At night, the bathymetry was retained as a fixed effect in the final model.

**Table 3:** Parameters estimation for the second model for days (DBD ∼ WMTH + temp200+ Light150) with standard error and p-values.

	Estimated coefficient	Standard error	p-value
Water Masses Temperature Heterogeneity (WMTH)	4.31	0.18	<0.001
Temperature at 200 m	17.11	0.86	<0.001
Light at 150 m	4.33	0.07	<0.001

(DBD: Diving Bottom Depth.).

**Table 4:** Parameters estimation for the second model for nights (DBD ∼ WMTH + Bathymetry + Light at 150 m) with standard error and p-values.

	Estimated coefficient	Standard error	p-value
Water Masses Temperature Heterogeneity (WMTH)	6.32	1.67	<0.001
Bathymetry	0.08	0.02	<0.001
Light at 150 m	3.45	0.48	<0.001

(DBD: Diving Bottom Depth.).

## Discussion

### 1 Oceanography

Predicted values of chl-a concentration estimated from K_0.75_ attenuation coefficient were strongly correlated with the observed values, supporting our initial hypothesis that light attenuation provides a good proxy of *in situ* phytoplankton concentration. However, a large percentage (72%) of the total variance of chl-a concentration remains unexplained indicating that factors other than phytoplankton concentration alone are playing an active role. Particles in suspension in the water column (other than phytoplankton) can also contribute to light attenuation, e.g. inorganic particles like CDOM and also zooplankton. Furthermore, fluorescence in itself is only a proxy of phytoplankton concentration and fluorescence response is known to vary with phytoplankton species and physiological state [33]. In addition, phytoplankton species differ to a large extent in their size and shape. Therefore, for a given chl-a concentration estimated from the fluorometer, we can expect a variation in the light attenuation factor according to phytoplankton species. A comparison with data of high resolution measurements of ocean colour from PHYSAT, which distinguishes the dominant phytoplankton groups within a given area [41], could be used in the future to evaluate the effect of phytoplankton species on light attenuation. However, as coastal areas were excluded from our study, we believe that turbidity had a limited effect on the light attenuation coefficients we calculated.

Despite the fact that light attenuation only provides an imperfect proxy of phytoplankton concentration, such measurements are likely to improve our understanding of the horizontal phytoplankton structuring at the sub-mesoscale. Until now, only mesoscale features could be investigated using fluorescence. Indeed, up to 40 attenuation coefficients can be calculated daily along the track of a SES during summer daylight hours at Kerguelen latitude’s, and up to 60 for one day (24 daylighthours) when SES are foraging in high Antarctic waters during summer. Furthermore, in comparison to fluorescence measurements, light requires very small amount of electrical power to be measured and therefore can be collected over extended periods of time.

The mean K_0.75_ obtained in this study corresponds to a value characteristic of oligotrophic waters. This is in agreement with the literature suggesting that the SO is relatively biologically poor [42], and that overall productivity has to be related to extensive area rather than productivity per unit of surface. Seals were at sea during the austral spring and early summer and, therefore, encompass the bloom and maximum phytoplankton concentration period occurring over December [43]. The seasonal increase of phytoplankton concentration is supported by the positive relationship identified between K_0.75_ and time.

Phytoplankton concentration estimated from fluorescence or K_0.75_ was found to decrease with increasing temperature at 200 m as well as with the number of temperature inversions within the water column. This finding is consistent with the fact that higher phytoplankton concentrations are generally found in association with cyclonic eddies exhibiting colder cores than surrounding waters [44]. These colder cyclonic eddies have a southward origin and the upwelling generated within these mesoscale features injects nutrient rich water into the euphotic layer, stimulating primary production. Lower phytoplankton concentrations were found in locations characterized by vertical temperature heterogeneity. Such vertical heterogeneity, if coinciding with vertical density heterogeneity, could be indicative of the intensity of shearing processes and vertical mixing. At high intensities these two mechanisms are known to locally limit the primary productivity due to the lack of water column stability and a deeper mixed layer depth, diluting phytoplankton concentration and causing deep mixing of the biomass [45]. In the present work, the reconstruction of chl-a profiles from light measurements [27] was not performed as this was not the main objective of this study. However, this will be investigated in future work and the accuracy of such reconstructions will be assessed by concomitant chl-a profiling provided by the fluorometer.

### 2 Ecology

Many studies have been able to relate oceanographic conditions to the prey availability and to predators [8], [10], [15], [17], [19], [22], [23], [46].However, to our knowledge, this is the first study showing that light level within the water column is a critical determinant of diving depth of SES. During daylight hours nearly sixty percent of the variance in daylight DBD of SES could be explained by the light level at 150 m. Previous studies have shown that moonlight affects the diving depth of Galapagos fur seals, with these mammals diving deeper during full moon nights compared to moonless nights [30]. Several studies have shown that light level precisely controls the vertical distribution of many species of crustaceans as well as myctophid fish.

By day and by night, water surface light level is likely to be related to abiotic factors such as cloud cover. During daylight hours, sun angle also plays a significant role. However, phytoplankton concentration is the critical factor controlling light attenuation and, therefore, light levels below the euphotic layer. Our finding is consistent with the result obtained by Dragon et al. [25]showing that diving depths of SES were negatively related to remotely sensed surface chl-a concentration. This result was interpreted as a possible effect of reduced light within the water column, allowing prey to be closer to the water surface and therefore more accessible to the diving seals. Our results confirm this interpretation, and show that this phenomenon is not limited to daylight hours. Myctophids, a major component of the female elephant seal's diet, are known to perform diurnal vertical migrations in response to surface light conditions [31]. The extent to which light affected the depth distributions of SES prey species was unexpected. Indeed, when light penetrated to as deep as 150 m – presumably representative of bright, sunny conditions and clear water – SES reached depths exceeding 700 m.

Interestingly, during daylight hours, at the bottom phase of the seal dive, a particular light intensity was measured, slightly above the detection level of the TDR light sensor (42.1±13.7 which corresponds to less than 10^−10^ W.cm^−2^). This light level could be indicative of the light optimum of their prey species, such as myctophids.

While light is a dominating factor, for a given light level, SES were found to dive deeper in locations where water was warmer and the vertical water temperature heterogeneity was greater. This suggests that other factors contribute to controlling the vertical distribution of SES prey. The positive effect of bathymetry on the DBD at night remains unclear to us, and this result will need to be confirmed by further investigations.

The relationship between temperature and diving depth has been emphasized by previous studies [22], [25]. Our work reveals that the effect of these factors is particularly strong at night, but that light is the key factor during daylight hours. These results are consistent with previous studies showing that vertical distribution of myctophidsis highly related to temperature [31], [47], [48]. However the role of light in controlling the vertical distribution of prey species is defined by a combination of light and temperature. Other parameters, such as dissolved oxygen, known to control the vertical distribution of a broad range of marine organisms,arelikely to be contributing factors. It is less clear to us how vertical temperature heterogeneity may act, but this could be indicative of areas of lower prey abundance, with elephant seals performing more exploratory dives. These dives are known to be deeper under such conditions [49].

Within the polar frontal zone, chl-a concentration was found to vary by factor of two between years of high primary production and years of low primary production [41]. Primary production is likely to impact upon the richness of secondary production and, therefore, prey abundance for secondary and apex predators. Furthermore, during years of low phytoplankton concentration, light attenuation is likely to be lower and SES may therefore have to dive deeper and expend more energy to access prey. By extension, a decrease in phytoplankton concentration may not only impact SES through prey availability, but also through vertical accessibility. These factors would result in increasing foraging costs and a decrease in foraging and demographic performances. This effect should be taken into account when investigating the possible ecological consequences on deep diving predators in relation to climate change.

## References

[1] Gould WJ, Turton J (2006) Argo–sounding the oceans. Weather 61: 17–21.

[2] Roquet F, Park YH, Guinet C, Bailleul F, Charrassin JB (2009) Observations of the Fawn Trough Current over the Kerguelen Plateau from instrumented elephant seals. Journal of Marine Systems 78: 377–393.

[3] Klatt O, Boebel O, Fahrbach E (2007) A profiling float's sense of ice. Journal of Atmospheric and Oceanic Technology 24: 1301–1308.

[4] Block BA, Costa DP, Boehlert GW, Kochevar RE (2002) Revealing pelagic habitat use: the tagging of Pacific pelagics program. Oceanologica Acta 25: 255–266.

[5] Eckert SA, Stewart BS (2001) Telemetry and satellite tracking of whale sharks, Rhincodon typus, in the Sea of Cortez, Mexico, and the north Pacific Ocean. Environmental Biology of Fishes 60: 299–308.

[6] Sims DW, Southall EJ, Richardson AJ, Reid PC, Metcalfe JD (2003) Seasonal movements and behaviour of basking sharks from archival tagging: no evidence of winter hibernation. Marine Ecology Progress Series 248: 187–196.

[7] Weimerskirch H, Catard A, Prince PA, Cherel Y, Croxall JP (1999) Foraging white-chinned petrels Procellaria aequinoctialis at risk: from the tropics to Antarctica. Biological Conservation 87: 273–275.

[8] Boyd I, Arnbom T (1991) Diving behaviour in relation to water temperature in the southern elephant seal: foraging implications. Polar Biology 11: 259–266.

[9] McConnell B, Chambers C, Fedak M (1992) Foraging ecology of southern elephant seals in relation to the bathymetry and productivity of the Southern Ocean. Antarctic Science 4: 393–393.

[10] Croxall J, Everson I, Kooyman G, Ricketts C, Davis R (1985) Fur seal diving behaviour in relation to vertical distribution of krill. The Journal of Animal Ecology: 1–8.

[11] Boehlert GW, Costa DP, Crocker DE, Green P, O Brien T, et al (2001) Autonomous pinniped environmental samplers: using instrumented animals as oceanographic data collectors. Journal of Atmospheric and Oceanic Technology 18: 1882–1893.

[12] Hooker SK, Boyd IL (2003) Salinity sensors on seals: use of marine predators to carry CTD data loggers. Deep Sea Research Part I: Oceanographic Research Papers 50: 927–939.

[13] Lydersen C, Anders Nøst O, Kovacs KM, Fedak MA (2004) Temperature data from Norwegian and Russian waters of the northern Barents Sea collected by free-living ringed seals. Journal of Marine Systems 46: 99–108.

[14] Boehme L, Lovell P, Biuw M, Roquet F, Nicholson J, et al.. (2009) Technical Note: Animal-borne CTD-Satellite Relay Data Loggers for real-time oceanographic data collection. Ocean Science.

[15] Biuw M, Boehme L, Guinet C, Hindell M, Costa D, et al (2007) Variations in behavior and condition of a Southern Ocean top predator in relation to in situ oceanographic conditions. Proceedings of the National Academy of Sciences 104: 13705. 10.1073/pnas.0701121104 PMC1959446 17693555

[16] Charrassin JB, Hindell M, Rintoul S, Roquet F, Sokolov S, et al (2008) Southern Ocean frontal structure and sea-ice formation rates revealed by elephant seals. Proceedings of the National Academy of Sciences 105: 11634. 10.1073/pnas.0800790105 PMC2575336 18695241

[17] McConnell B, Fedak M (1996) Movements of southern elephant seals. Canadian Journal of Zoology 74: 1485–1496.

[18] Lewis R, O'Connell TC, Lewis M, Campagna C, Hoelzel AR (2006) Sex-specific foraging strategies and resource partitioning in the southern elephant seal (Mirounga leonina). Proceedings of the Royal Society B: Biological Sciences 273: 2901–2907. 17015314 10.1098/rspb.2006.3642 PMC1664629

[19] Bailleul F, Cotté C, Guinet C (2010) Mesoscale eddies as foraging area of a deep-diving predator, the southern elephant seal. Marine Ecology-Progress Series 408: 251–264.

[20] Bailleul F, Charrassin JB, Ezraty R, Girard-Ardhuin F, McMahon CR, et al (2007) Southern elephant seals from Kerguelen Islands confronted by Antarctic Sea ice. Changes in movements and in diving behaviour. Deep Sea Research Part II: Topical Studies in Oceanography 54: 343–355.

[21] Cherel Y, Ducatez S, Fontaine C, Richard P, Guinet C (2008) Stable isotopes reveal the trophic position and mesopelagic fish diet of female southern elephant seals breeding on the Kerguelen Islands. Marine Ecology Progress Series 370: 239–247.

[22] Bailleul F, Charrassin JB, Monestiez P, Roquet F, Biuw M, et al (2007) Successful foraging zones of southern elephant seals from the Kerguelen Islands in relation to oceanographic conditions. Philosophical Transactions of the Royal Society B: Biological Sciences 362: 2169–2181. 10.1098/rstb.2007.2109 PMC2442861 17472917

[23] Field I, Hindell M, Slip D, Michael K (2001) Foraging strategies of southern elephant seals (Mirounga leonina) in relation to frontal zones and water masses. Antarctic Science 13: 371–379.

[24] Bradshaw CJA, Higgins J, Michael KJ, Wotherspoon SJ, Hindell MA (2004) At-sea distribution of female southern elephant seals relative to variation in ocean surface properties. ICES Journal of Marine Science: Journal du Conseil 61: 1014–1027.

[25] Dragon AC, Monestiez P, Bar-Hen A, Guinet C (2010) Linking foraging behaviour to physical oceanographic structures: Southern elephant seals and mesoscale eddies east of Kerguelen Islands. Progress in Oceanography 87: 61–71.

[26] Roquet F, Charrassin JB, Marchand S, Boehme L, Fedak M, et al.. (2011) Delayed-mode calibration of hydrographic data obtained from animal-borne satellite-relay data loggers. Journal of Atmospheric and Oceanic Technology.

[27] Teo SLH, Kudela RM, Rais A, Perle C, Costa DP, et al (2009) Estimating chlorophyll profiles from electronic tags deployed on pelagic animals. Aquatic Biology 5: 195–207.

[28] Liu SH, Sun S, Han BP (2003) Diel vertical migration of zooplankton following optimal food intake under predation. Journal of plankton research 25: 1069–1077.

[29] Batty R, Blaxter J, Richard J (1990) Light intensity and the feeding behaviour of herring, Clupea harengus. Marine Biology 107: 383–388.

[30] Horning M, Trillmich F (1999) Lunar cycles in diel prey migrations exert a stronger effect on the diving of juveniles than adult Gal pagos fur seals. Proceedings of the Royal Society of London Series B: Biological Sciences 266: 1127–1132. 10406130 10.1098/rspb.1999.0753 PMC1689955

[31] Catul V, Gauns M, Karuppasamy P (2011) A review on mesopelagic fishes belonging to family Myctophidae. Reviews in Fish Biology and Fisheries 21: 339–354.

[32] Bricaud A, Morel A, Babin M, Allali K, Claustre H (1998) Variations öf light absorption by suspended particles with chlorophyll a concentration in oceanic (case 1) waters: Analysis and implications for bio-optical models. Journal of Geophysical Research 103: 31,033–031,044.

[33] Xing X, Claustre H, Blain S, D'Ortenzio F, Antoine D, et al.. (2011) Quenching correction for in vivo chlorophyll fluorescence measured by instrumented elephant seals in the Kerguelen region. Limnology and Oceanography methods Submitted.

[34] Gordon HR (1989) Can the Lambert-Beer law be applied to the diffuse attenuation coefficient of ocean water? Limnology and Oceanography: 1389–1409.

[35] Team RDC (2009) R: A language and environment for statistical computing. In: Computing RFfS, editor.

[36] Laird NM, Ware JH (1982) Random-effects models for longitudinal data. Biometrics: 963–974. 7168798

[37] Ichiye T (1967) Occurrence of temperature inversions in the upper layer of the ocean. Pure and Applied Geophysics 67: 143–155.

[38] Hense I, Bathmann UV, Timmermann R (2000) Plankton dynamics in frontal systems of the Southern Ocean. Journal of Marine Systems 27: 235–252.

[39] Nelson DM, Smith Jr WO (1991) Sverdrup revisited: critical depths, maximum chlorophyll levels, and the control of Southern Ocean productivity by the irradiance-mixing regime. Limnology and Oceanography: 1650–1661.

[40] Rasmus K, Granéli W, Wängberg SÅ (2004) Optical studies in the Southern Ocean. Deep Sea Research Part II: Topical Studies in Oceanography 51: 2583–2597.

[41] Alvain S, Moulin C, Dandonneau Y, Bréon FM (2005) Remote sensing of phytoplankton groups in case 1 waters from global SeaWiFS imagery. Deep Sea Research Part I: Oceanographic Research Papers 52: 1989–2004.

[42] Kirk JTO (1994) Light and photosynthesis in aquatic ecosystems: Cambridge Univ Pr.

[43] Dragon AC, Bar-Hen A, Monestiez P, Guinet C (2012) Horizontal and vertical movements as predictors of foraging success in a marine predator. Mar Ecol Prog Ser 447: 243–257.

[44] Kahru M, Mitchell B, Gille S, Hewes C, Holm-Hansen O (2007) Eddies enhance biological production in the Weddell-Scotia Confluence of the Southern Ocean. Geophys Res Lett 34: L14603.

[45] Lévy M (2008) The modulation of biological production by oceanic mesoscale turbulence. Transport and Mixing in Geophysical Flows: 219–261.

[46] Campagna C, Piola AR, Rosa Marin M, Lewis M, Fernández T (2006) Southern elephant seal trajectories, fronts and eddies in the Brazil/Malvinas Confluence. Deep Sea Research Part I: Oceanographic Research Papers 53: 1907–1924.

[47] Hulley PA (1981) Results of the Research Cruises of FRV" Walther Herwig" to South America: LVIII. Family Myctophidae (Osteichthyes, Myctophiformes): Heenemann.

[48] Loots C, Koubbi P, Duhamel G (2007) Habitat modelling of Electrona antarctica (Myctophidae, Pisces) in Kerguelen by generalized additive models and geographic information systems. Polar Biology 30: 951–959.

[49] Dragon AC, Bar-Hen A, Monestiez P, Guinet C (2012) Horizontal area-restricted-search and vertical diving movements to predict foraging success in a marine predator.

